# Development of a Highly Adaptive Miniature Piezoelectric Robot Inspired by Earthworms

**DOI:** 10.1002/advs.202403426

**Published:** 2024-06-05

**Authors:** Jie Deng, Ziteng Liu, Jing Li, Shijing Zhang, Yingxiang Liu

**Affiliations:** ^1^ State Key Laboratory of Robotics and System Harbin Institute of Technology Harbin Heilongjiang 150001 China

**Keywords:** high adaptability, longitudinal vibrations, miniature robots, piezoelectric driving, resonant actuation

## Abstract

Miniature resonant piezoelectric robots have the advantages of compact structure, fast response, high speed, and easy control, which have attracted the interest of many scholars in recent years. However, piezoelectric robots usually suffer from the problem of poor adaptability due to the micron‐level amplitude at the feet. Inspired by the fact that earthworms have actuation trajectories all around their bodies to move flexibly under the ground, a miniature piezoelectric robot with circumferentially arranged driving feet to improve adaptability is proposed. Notably, a longitudinal‐vibration‐compound actuation principle with multilegged collaboration is designed to achieve the actuation trajectories around the robot, similar to the earthworms. The structure and operating principle are simulated by the finite element method, and the prototype is fabricated. The robot weighs 22.7 g and has dimensions of 35.5 × 36.5 × 47 mm^3^. The robot is tethered to an ultrasonic power supply, and the experimental results show that the speed reaches 179.35 mm s^−1 ^ under an exciting signal with a frequency of 58.5 kHz and a voltage of 200 V_p‐p_. High adaptability is achieved by the proposed robot, it can move on flat, fold, concave, and convex surfaces, and even in an inclined or rotating tube.

## Graphical abstract

A miniature piezoelectric robot inspired by earthworms is proposed, and the principle of longitudinal‐vibration‐compound actuation with multilegged collaboration is designed to improve surface adaptability. The experimental results show that the robot achieves miniature size, lightweight, and high adaptability. The robot can adapt to flat, fold, concave, and convex surfaces, and even incline and dynamically rotating tubes.

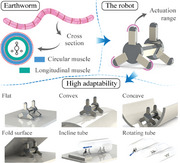

## 1 Introduction

Miniature mobile robots have aroused great interest among researchers because of their broad application prospects, such as surveillance, exploration, search and rescue operations, and so on.^[^ ^1^, ^2^, ^3^, ^4^ ^]^ Compared with the traditional middle and large robots, miniature robots possess the inherent advantages of miniature size, lightweight and flexible movement, and they can be easily transported and deployed in hard‐to‐reach environments. Typically, actuators are the most critical component that enables the motion capacity. The traditional electromagnetic actuator with an appropriate structure design can be used for miniature mobile robots.^[^ ^5^, ^6^ ^]^ However, their motion characteristics and structure sizes are hard to balance due to the transmission system, which limits their application in the above scenarios.

In recent decades, significant advancements have been made in the development of actuators using smart materials, which have largely overcome the limitations of traditional electromagnetic actuators. Various types of actuators have been introduced, including shape memory alloy actuators (SMAs), electrostrictive actuators (EAs), magnetostrictive actuators (MAs), and piezoelectric actuators (PAs). SMAs are mainly thermally activated without transmission mechanisms, so they are ideal actuators for the miniaturization design. However, the output speed of the SMAs is low due to the slow temperature response, which limits their application in high‐speed scenes.^[^ ^7^, ^8^ ^]^ EAs have the advantages of lightweight and high energy density, but the high actuating voltage limits their further application in miniature mobile robots.^[^ ^9^, ^10^ ^]^ MAs are well known for their high efficiency and long service life, but they work in high‐intensity magnetic fields, the application scenarios are also limited.^[^ ^11^, ^12^ ^]^ Compared with these actuators, the PAs are particularly attractive because of their simple structure, fast response, large output force, and excellent controllability,^[^ ^13^, ^14^, ^15^, ^16^, ^17^, ^18^ ^]^ and a number of miniature robots with PAs have been developed.^[^ ^19^, ^20^, ^21^, ^22^ ^]^

According to the working mode, miniature piezoelectric robots can be divided into non‐resonant type and resonant type. For the non‐resonant type robots, the piezoelectric actuators operate in the non‐resonant frequency range, precision motion can be achieved.^[^ ^23^, ^24^ ^]^ Moon et al. developed an inchworm piezoelectric robot,^[^ ^25^ ^]^ linear motion was achieved on the guide rail, obtaining 50 nm accuracy and a maximum speed of 10.2 mm s^−1^ was obtained. Fuchiwaki et al. designed an insect‐sized holonomic robot that can move in any direction in an inchworm fashion,^[^ ^26^ ^]^ with a size of 32 × 32 × 20 mm, achieving a high resolution of 25 nm and a maximum speed of about 1.4 mm s^−1^. Zhong et al. proposed a precision motion robot based on the inertial stick‐slip driving principle,^[^ ^27^ ^]^ the dimension is 10 × 10 × 10 mm, a maximum speed of 13.1 mm s^−1^ and a resolution of 20 nm achieved by direct deformation of its inertial unit. It can be concluded that the motion speed and working surface adaptability are poor, this is because the output displacement of the piezoelectric actuator is at the micro‐nano level and the operating frequency is low. Although the output speed can be improved by increasing the output displacement, it can also lead to an increase in the robot size.

The resonant‐type miniature piezoelectric robots can alleviate the above problems, its piezoelectric actuator operates in the resonant frequency with large vibration amplitude, which is helpful to achieve high motion speed and good working surface adaptability.^[^ ^28^, ^29^, ^30^ ^]^ Wood et al. developed a resonant quadrupedal bionic robot HAMR‐F,^[^ ^31^ ^–^ ^34^ ^]^ The length and the mass were only 45 mm and 2.8 g, respectively, and it can move in a plane with a speed of 172 mm s^−1^. Rios et al. developed a miniature six‐legged piezoelectric robot MinRAR V2.^[^ ^35^, ^36^ ^]^ The robot integrates a miniature control system and a power supply and can use external power to excite motion as well as its own power to excite motion, achieving a maximum motion speed of 98 mm s^−1^ at an excitation frequency of 190 Hz. In addition to the above robots with modal composite actuation,^[^ ^37^ ^]^ there are some robots driven by traveling or standing waves.^[^ ^38^ ^]^ Wang et al. proposed a novel ring‐shaped tripodal piezoelectric robot by standing and traveling wave with a weight of 2.2 g and a size of 20 × 20 × 4.6 mm and achieves a maximum speed of 39.3 mm s^−1^.^[^ ^39^ ^]^ Li et al. designed a novel miniature tripodal piezoelectric robot,^[^ ^40^ ^]^ that can excite various standing waves by a specific arrangement of PZT sheets, with a size of Φ67 × 27 mm^3^ and a maximum speed of 231.61 mm s^−1^. Jia et al. proposed a traveling wheel piezoelectric robot,^[^ ^41^ ^]^ in which the wheel structure is driven by a traveling wave, with a size of 148.6 × 28.6 × 11 mm, a maximum no‐load speed of 136.8 mm s^−1^, and a prototype achieved 75° step climbing. In summary, the motion speed and working surface adaptability can be improved by operating in resonant frequency for miniature piezoelectric robots. However, the adaptability of the existing resonant‐type miniature piezoelectric robots is still inadequate, they usually work on a static flat surface, and cannot work on concave and convex surfaces, especially in dynamically moving curved surface, such as in a rotating tube, which limits the application in tube exploration and search.

**Figure 1:**
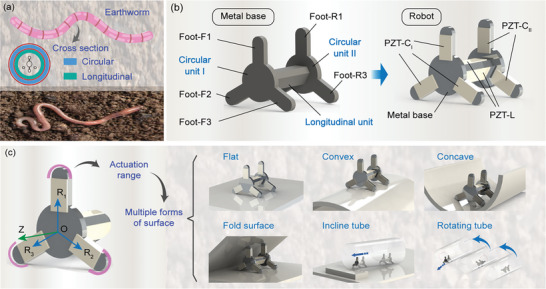
Structural and highly adaptable display of the robot. a) Diagram of an earthworm and its circular and longitudinal muscles. b) Main structure of the robot. c) Demonstration of high adaptability, including flat, fold, concave, and convex surfaces, and even incline and dynamically rotating tubes.

Interestingly, we noticed that earthworms can move flexibly in complex underground environments. In addition to their soft bodies, another advantageous factor is that earthworms have actuation trajectories all around their bodies, generated by their circular and longitudinal muscles,^[^ ^42^ ^]^ as shown in **Figure** **1**a. Inspired by the around‐body actuation trajectories of earthworms, we proposed a resonant miniature piezoelectric robot with circumferentially arranged driving feet to improve adaptability, which also achieved a simple structure, a high speed, and a lightweight. Specifically, the proposed piezoelectric robot realized the following advancements: i) with reference to the muscles of earthworms, a longitudinal‐vibration‐compound actuation principle was proposed to generate the actuation trajectories around the robot body; ii) a separate and circumferential arrangement of driving feet was designed, which help the robot adapt to other complex situations outside a plane, such as concave and convex surfaces; iii) a robot prototype with a length of 35.5 mm and a weight of 22.7 g was fabricated, which achieved a speed of 179.35 mm s^−1 ^ at a resonant working frequency of 58.5 kHz and avoided the working noisy; iv) the surface adaptability was investigated, the robot could adopt not only to planes of different roughness, but also to fold surfaces, concave surfaces, convex surfaces, and even dynamically rotating tube.

## 2 Results

### 2.1 Configuration Design

Generally, the working surfaces of a miniature robot include the following types: flat surface, fold surface, concave surface, and convex surface. For adapting to the various working surfaces, a miniature piezoelectric robot is proposed with a unique feet arrangement circumferentially and separately, as shown in Figure 1b. This robot is mainly composed of a metal base and fifteen PZT (Lead Zirconium Titanate) sheets. The metal base is a symmetrical integrated structure, which not only strengthens the overall stiffness, but also simplifies the overall structural design, and the miniaturization of the robot is facilitated. For convenience of expression, it can be divided into 3 parts, named Circular units I, II, and Longitudinal units, respectively. Circular units I and II have exactly the same axially symmetrical structure, including a central connecting disc and their driving feet around the disc. According to the motion direction, the feet are named Foot‐F1, 2, 3 (forward) and Foot‐B1, 2, 3 (backward), respectively. The longitudinal unit is a triangular prism structure, and its 3 main surfaces are perpendicular to Foot‐F1, 2, and 3, respectively. For the PZT sheets, twelve sheets are pasted on the twelve sides of Foot‐Fs and Foot‐Bs, named PZT‐C_I_ and PZT‐C_II_, respectively; and the other 3 PZT sheets are pasted on the 3 main surfaces of the Longitudinal unit, named PZT‐L. Similar to the circular and longitudinal muscles of earthworms, PZT‐C_I, II,_ and PZT‐L are used to generate the vibration deformations of Circular units I, II, and Longitudinal units, respectively. Then, the vibrations of Circular units and Longitudinal units can synthesize elliptical trajectories at the driving feet (the specific synthesis principle is introduced in the next section). Notably, the entire periphery of the driving feet will produce actuation trajectories, as shown in Figure 1c. The entire‐periphery actuation trajectories, together with the circumferential distributed driving feet, can help the robot adapt to a variety of surfaces, including, flat, fold, concave, and convex surfaces, and even incline and dynamically rotating tubes (see Movie S1, Supporting Information).

### 2.2 Working Principle

**Figure 2:**
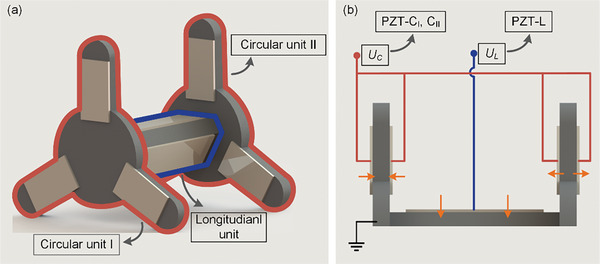
The unit partition of the robot and the layout and polarization direction of the PZT sheets. a) The component units of the robot. b) Circuit connections of the robot and the layout and polarization direction of the PZT sheet.

**Figure 3:**
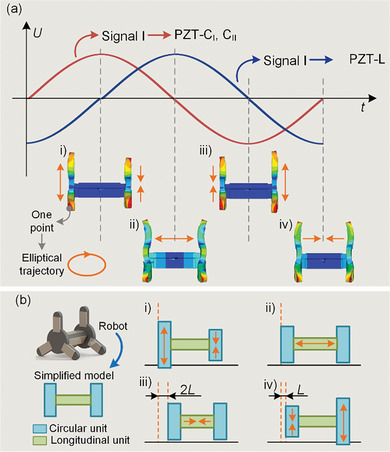
Operating principle of the robot. a) Units generate an elliptical trajectory under 2 sets of AC voltages with a 90° phase difference. b) The simplified models show that the robot moves in a cycle to obtain a displacement of *L*.

Circular units I, II, and longitudinal units with PZT sheets are shown in **Figure** **2**a, in which the PZT sheets use d_31_ mode to excite each unit to generate longitudinal vibration. The designed excitation scheme is shown in Figure 2b, the exciting signal *U_C_* is applied to PZT‐C_I, II_ to control the longitudinal vibrations of the 2 Circular units, and the exciting signal *U_L_* is applied to the PZT‐L controls the longitudinal vibration of the Longitudinal unit. The direction of the yellow arrows represents the polarization direction of the PZT sheets. The used exciting signals and vibration modes of the robot in one cycle are shown in **Figure** **3**a. There is a 90° phase shift between the 2 signals, and an elliptical trajectory on the tip of the foot can be generated by the combination of the longitudinal vibrations of Circular and Longitudinal units. In order to facilitate understanding, we simplify the movement process of the robot, as shown in Figure 3b, in which the specific operation in one cycle can be divided into 4 stages.

In stage i), Circular unit II shortens and the foot moves in the positive direction of the y‐axis, the Longitudinal unit returns to the original length and the foot feet moves along the x‐axis in the positive direction;

In stage ii), Circular unit II returns to the original length, and the foot moves in the opposite direction of the y‐axis, The Longitudinal unit extends to push the foot in the positive direction of the x‐axis;

In stage iii), Circular unit II extends and the foot moves further along the y‐axis in the opposite direction, the Longitudinal unit returns to the original length, and the foot moves along the x‐axis in the opposite direction;

In stage iv), Circular unit II returns to the original length, the foot moves again in the positive direction of the y‐axis, and the Longitudinal unit shortens to drive the foot to move along the x‐axis in the opposite direction.

As a result, the foot returns to stage i) and completes a whole cycle, in which an elliptical trajectory in a clockwise direction is generated at the top point of the foot. The friction between the foot and the working surface drives the robot to move forward. At the same time, the foot of Circular unit I performs a cycle of iii)‐iv)‐i)‐ii), which also produces an elliptical trajectory in a clockwise direction. Moreover, the reverse motion can be achieved by exchanging the 2 signals applied to Circular unit I and Circular unit II. As a result, the robot can move step‐by‐step based on the friction between the feet and the working surface.

### 2.3 Simulation Analysis and Parameters Determination

Simulation analyses are performed to determine the structure and material parameters of the robot, and the design goals are determined by the actuation principle: 1) the amplitude of the foot should be on the micrometer scale to leave away from the working surface; 2) the Circular units and Longitudinal unit should operate in longitudinal vibration modes to achieve the extension and shorten actions, and the corresponding operating frequencies should be consistent and in the ultrasonic frequency band; 3) the size should be designed as miniature as possible to achieve movement in a narrow space.

First, the modal analyses are carried out to investigate the longitudinal vibration modes and the corresponding resonance frequencies of the Circular units and Longitudinal units. The hard aluminum alloy 2A12 is used for the metal structure and PZT‐4 (Hongsheng Acoustic Electronic Equipment Co., Baoding) is chosen for the PZT sheets. The material parameters are shown in **Table** **1**.

**Table 1:** Material parameters of metal base and PZT sheets.

Materials	Density [kg m^−3^]	Young's modulus [Pa]	Piezoelectric constant [C m^−2^]	Poisson's ratio
Aluminum	2700	72 × 10^9^	/	0.3
PZT‐4	7600	$\left[\begin{array}{cccccc} 14.3 & 7.85 & 7.85 & 0 & 0 & 0\\ 7.85 & 14.3 & 7.85 & 0 & 0 & 0\\ 7.85 & 7.85 & 11.5 & 0 & 0 & 0\\ 0 & 0 & 0 & 2.6 & 0 & 0\\ 0 & 0 & 0 & 0 & 2.45 & 0\\ 0 & 0 & 0 & 0 & 0 & 2.45 \end{array}\right]\times 10^{10}$	$\left[\begin{array}{ccc} 0 & 0 & -2.4\\ 0 & 0 & -2.4\\ 0 & 0 & 17.3\\ 0 & 0 & 0\\ 0 & 12.95 & 0\\ 12.95 & 0 & 0 \end{array}\right]$	/

**Figure 4:**
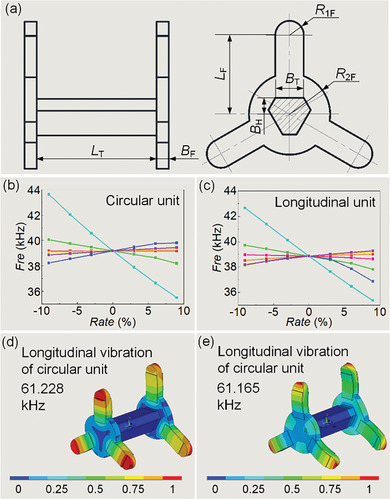
Analysis of the structural parameters of the robot. a) Main dimensional parameters of the metal base. b) Parameter sensitivity analysis results of circular unit. c) Parameter sensitivity analysis results of longitudinal unit. d) Longitudinal vibration of the circular unit. e) Longitudinal vibration of the longitudinal unit (The scale has been normalized).

The main dimensional parameters are shown in **Figure** **4**a, and the parametric sensitivity analyses of the longitudinal vibration modal frequencies are performed. The influences of each parameter on the modal frequencies of the Circular units and Longitudinal unit are shown in Figure 4b,c, respectively. It can be found that the length of the foot (L*_F_*) has the greatest influence on the modal frequencies, it can be used to adjust the total dimension of the robot. It is possible to achieve consistent modal frequencies of the Circular units and Longitudinal units in the ultrasonic frequency band by adjusting the parameters according to the parameter sensitivity results. The adjusted robot structure parameters are shown in **Table** **2**, and the dimensions of the PZT sheet are 24 × 7 × 0.5 mm for Longitudinal units and 12 × 7 × 0.5 mm for Circular units. The longitudinal vibration frequency of the Circular units is 61.23 kHz, as shown in Figure 4d, and the longitudinal vibration frequency of the Longitudinal unit is 61.17 kHz, as shown in Figure 4e. The resonance frequency difference among them is about 60 Hz, with a relative difference of approximately 0.1%, which is within the allowable range of modal simplicity error.

**Table 2:** Parameters of robot metal structure.

Parameter Name	Symbol	Length [mm]	Parameter Name	Symbol	Length [mm]
Length of the foot of the circular unit	*L*_F_	19.5	Length of Longitudinal unit	*L*_T_	29.5
The radius of the foot of the circular unit	*R*_1F_	3.5	Width of Longitudinal unit	*B*_T_	7.0
The radius of the circular unit disc	*R*_2F_	10.0	Height of Longitudinal unit	*H*_T_	4.0
Width of circular unit	*B*_F_	3.0			

Then, transient analyses are carried out to investigate the theoretical vibration amplitudes and trajectories of the feet. The transient simulation analyses mainly include the following 2 aspects: 1) vibration coupling analysis; the exciting signal is applied to the Circular units or Longitudinal unit, and the coupling vibration of the other units can be obtained. For example, the coupling vibrations of the Circular units are generated when the Longitudinal unit is excited. 2) overall analysis; the Longitudinal and Circular units are excited simultaneously; the vibration amplitudes and trajectories of the feet can be obtained.

**Figure 5:**
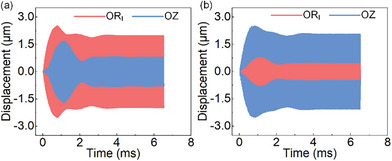
Vibration coupling analyses at the driving foot. a) The vibration amplitudes when only exciting PZT‐C_I_, C_II_. b) The vibration amplitudes when only exciting PZT‐L.

The vibration coupling analyses are carried out by applying voltage to the units individually. The voltage amplitude and frequency of the signals are set as 100 V and 61.20 kHz which is the average frequency of the 2 longitudinal vibration frequencies, the displacement on the foot is extracted. As shown in **Figure** **5**a, the vibration amplitude along the Circular unit direction is 2.00 µm when only Circular units are excited, and the vibration amplitude along the Longitudinal unit direction is about 0.80 µm. Similarly, the vibration amplitudes along the Longitudinal and Circular unit directions are 1.90 and 0.45 µm when the Longitudinal unit is excited, as shown in Figure 5b. It can be found there is vibration coupling among the Circular units and Longitudinal units, which affects the symmetry of the elliptical trajectory of the foot.

**Figure 6:**
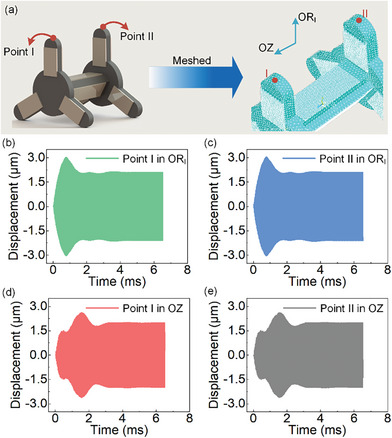
Overall transient simulation of the robot. a) Selection of suitable points on a pair of feet of the robot. b) Response displacements of point I in OR_I_. c) Response displacements of point II in OR_I_. d) Response displacements of point I in OZ. e) Response displacements of point II in OZ.

Two phases of signals are applied to Circular units and Longitudinal units simultaneously for the overall analyses. The voltage amplitude and frequency of the exciting signals are also set as 100 V and 61.20 kHz, and the initial phase difference of the 2 phases is 90°. The response displacements of points I and II are adopted to investigate the transient characteristics as the symmetrical structure, as shown in **Figure** **6**a, they are located at the top of the feet, respectively. As shown in Figure 6b–e, the response displacements of points I and II in 2 directions have good symmetry and the results are basically consistent. The data of the last cycle are extracted, the longitudinal vibration amplitude of Circular units is about 2.12 µm, and that of Longitudinal unit is about 2.00 µm.

**Figure 7:**
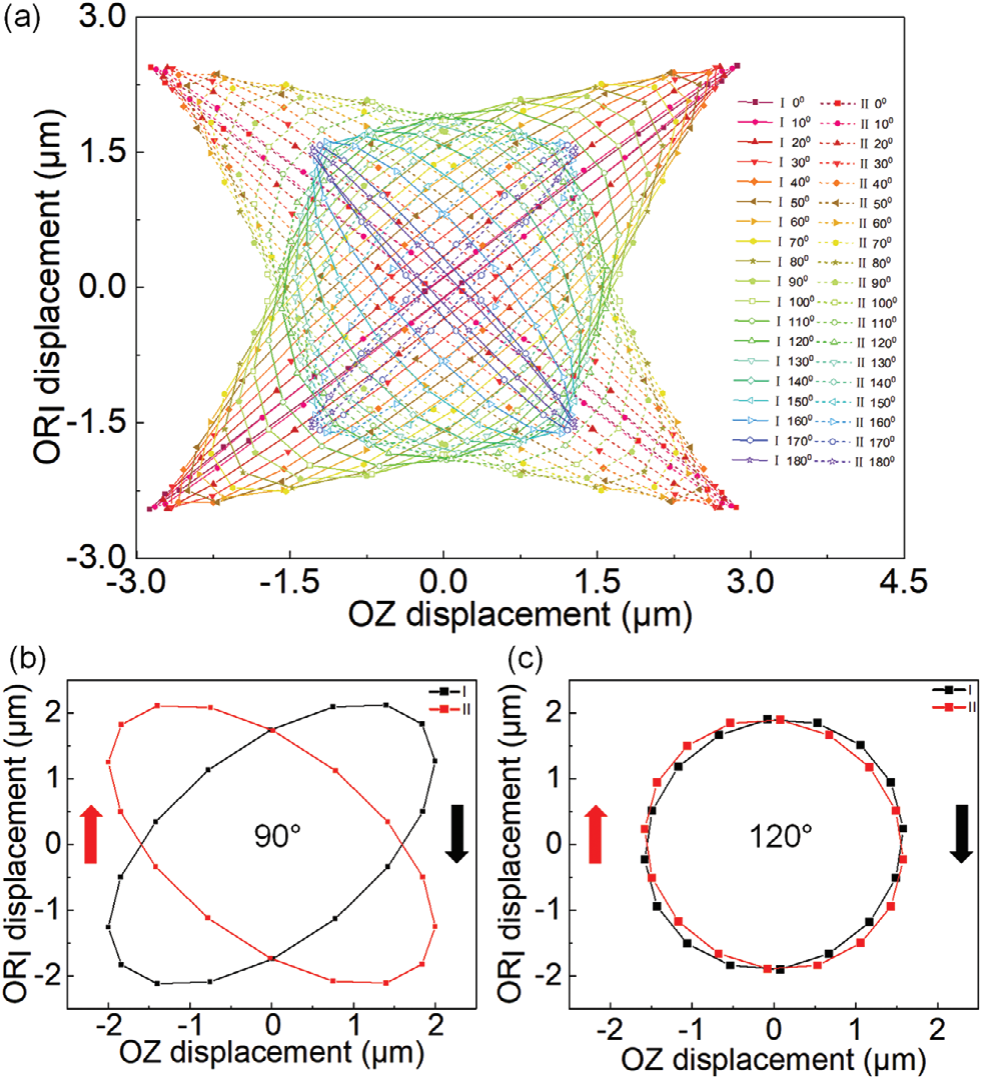
Trajectories of the feet tips. a) Trajectories at different phase differences. b) Trajectories at a phase difference of 90°. c) Trajectories at a phase difference of 120°.

In order to determine the optimum phase difference, the motion trajectories of Points I and II at different phase differences are investigated. Nineteen transient simulation analyses are carried out at the phase differences from 0° to 180°, and the results are shown in **Figure** **7**a. The motion trajectories at the phase difference of 90° are plotted, as shown in Figure 7b, and elliptical trajectories are successfully generated. It can be found that the generated trajectories are not positive ellipse, the phenomenon is caused by the vibration coupling among the Circular units and Longitudinal unit. While the trajectory approximates a positive ellipse when the phase difference is 120°, as shown in Figure 7c. The longitudinal vibration amplitude of the Longitudinal unit is about 1.8 µm, and that of the Circular units is about 1.6 µm, in which good motion performances can be achieved.

**Figure 8:**
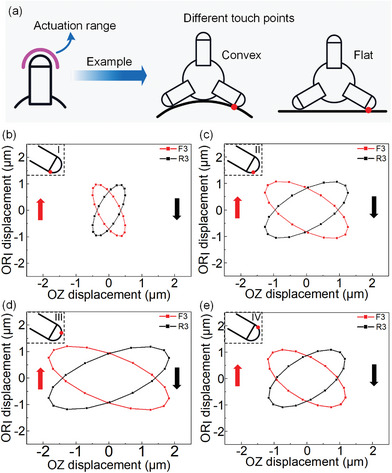
Trajectories of the robot feet on different surfaces. a) Selection of touch points for different surfaces from the actuation range. b) Trajectories at point I. c) Trajectories at point II. d) Trajectories at point III. e) Trajectories at point IV. (F3 indicates the trajectory of the touching point on FOOT‐F3 (see Figure 1b), and R3 indicates the trajectory of the touching point on FOOT‐R3).

Moreover, the trajectory data of the robot feet touching the flat and convex working surfaces are extracted, respectively, as plotted in **Figure** **8**a. It can be found that the elliptical trajectories can be formed on different working surfaces, and the speed of the robot on each working surface is different due to the different touch positions. 4 equally spaced points are taken on the surface of the driving foot, and the motion trajectories are shown in Figure 8b–d. It can be seen that the elliptical trajectory is larger as it gets closer to the top of the foot. As a result, the structural design goals of the robot are achieved, and the requirements of the actuation principle are satisfied: 1) the amplitudes of the foot in the Circular units and Longitudinal unit directions are 1.6 and 1.8 µm under the voltage amplitude of 100 V and frequency of 61.20 kHz, they are in the micrometer scale; 2) the Circular units and Longitudinal unit operate in longitudinal vibration modes and the longitudinal vibration frequencies are 61.23 and 61.17 kHz, they are consistent and in ultrasonic frequency band; 3) the robot has achieved a centimeter size with dimension of 35.5 × 36.5 × 47 mm.

### 2.4 Experiments of the Prototype

**Figure 9:**
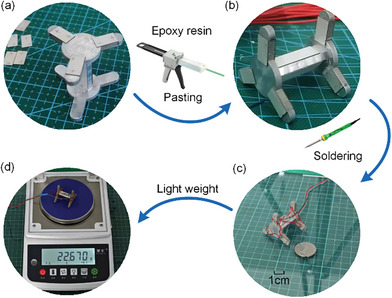
Prototype assembly process. a) Metal base and PZT sheets selection for the prototype. b) Bonding of metal base and PZT sheets using epoxy resin. c) Prototype after soldering wires and a coin. d) Prototype weight.

The robot prototype is assembled and fabricated according to the design and simulation results. The main prototype assembly steps are PZT sheets bonding and prototype wires welding, as shown in **Figure** **9**. The epoxy resin is used to bond the PZT sheets on the metal structure, and the wires are soldered to the PZT sheets on the metal structure as designed when the glue has been cured. Figure 9c shows the robot prototype and a coin, it can be found that the circumferential dimension is similar to that of a coin. The weight of the prototype is measured as 22.7 g, miniature size and light weight are achieved by the designed robot.

**Figure 10:**
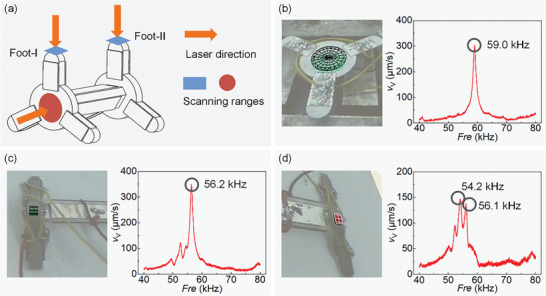
Vibration measurement results. a) Selection of the scanning ranges. b) Measurement result of the longitudinal unit. c) Measurement result of Foot‐I. d) Measurement result Foot‐II. (*V*_V_ indicates the vibration speed and *Fre* indicates the frequency).

The modal vibration modes and longitudinal vibration frequencies of the prototype are measured by a scanning laser vibrometer (PSV‐400‐M2, Polytec, Germany). The test areas are selected to measure the modal vibration modes of the Circular units and Longitudinal unit, as shown in **Figure** **10**a, where the blue test area is for measuring the longitudinal vibration of the Circular units and the red test area is for that of the Longitudinal unit. The test frequency range is set from 40 to 80 kHz. The measuring result of the Longitudinal unit is shown in Figure 10b, the longitudinal vibration frequency is 59.0 kHz, which has a certain gap with the simulation result of 61.2 kHz. Figure 10c,d show the results of the longitudinal vibrations of the feet on the 2 Circular units (Foot‐I and Foot‐II), respectively. The longitudinal vibration frequency of Foot‐I is 56.2 kHz, and the curve of Foot‐II has 2 peaks, corresponding to 54.2 and 56.1 kHz, respectively. The main reasons for these phenomena are machining and assembly errors, such as unsymmetrical structure, gluing, and wire welding errors.

**Figure 11:**
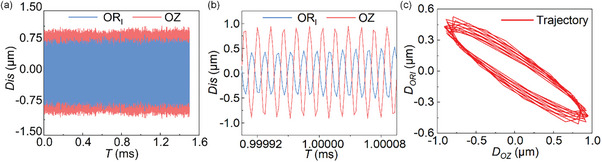
Displacement response characteristics measurement of a foot. a) Displacement curves in OR_I_ and OZ. b) Selection of a specific portion of the curves. c) Trajectory of the foot in the specific portion.

In order to further verify the actual vibration state of the prototype, 2 laser displacement sensors (LK‐H020, Keyence) are used to test the displacement response of one foot. The sampling frequency is set to 392 kHz to ensure that enough data points are collected in each cycle. The 2 laser displacement sensors are used to measure the displacement responses in Circular unit and Longitudinal directions, respectively, and the results are plotted in **Figure** **11**a, and the extracted data for 12 cycles is shown in Figure 11b. Then, the dynthetic trajectories of the 12 cycles at the foot tip are plotted in Figure 11c, it can be seen that the expected elliptical trajectories are successfully generated.

**Figure 12:**
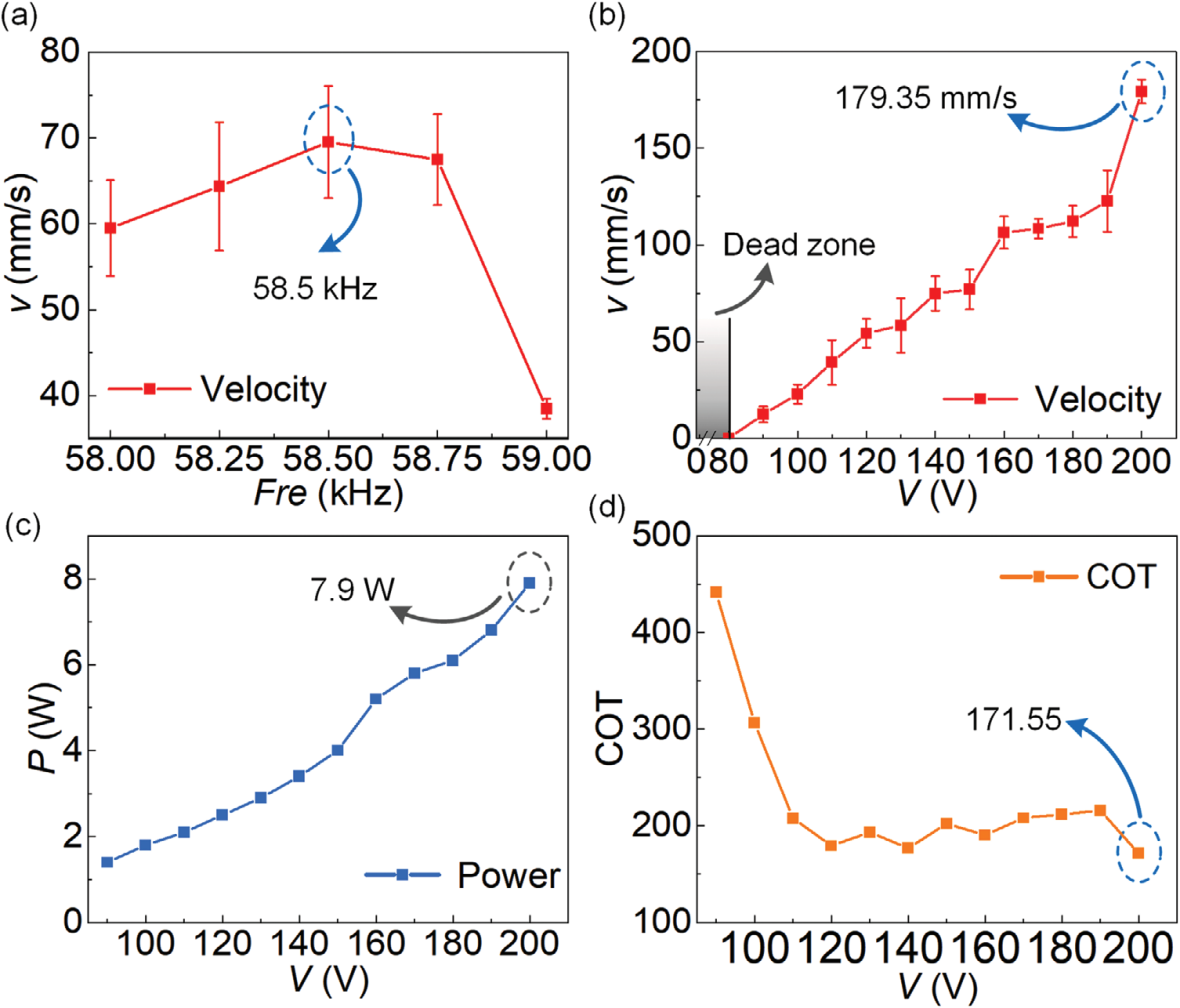
Experiments of the speed characteristics. a) Prototype speed versus frequency. b) Prototype speed versus voltage. c) Prototype power versus voltage. d) Cost of transportation versus voltage.

Next, the motion characteristics of the prototype are tested, mainly on the basic parameters of the prototype motion and the adaptability of the prototype to different surfaces. The main experiments are as follows: 1) the motion speed of the prototype at different frequencies and voltages; 2) the motion test of the prototype on working surfaces of different materials; 3) the motion test of the prototype on working surfaces of different shapes. In addition, demonstration experiments would be conducted for the prototype to verify its adaptability to complex environments. A camera is used to record the motion of the prototype. The speed of the robot is tested under signals with different frequencies; the voltage amplitude is set as 150 V_p‐p_ and the material of the flat working surface is marble. The result is shown in **Figure** **12**a, the fastest movement speed of 69.50 mm s^−1 ^ is achieved at the frequency of 58.5 kHz. Then the frequency is set to 58.5 kHz, and the motion speeds are tested under different voltages. The voltage test range is from 0 V_p‐p_ to 200 V_p‐p_, and the test result is shown in Figure 12b. The speed gradually increases as the voltage increases. The motion speed is close to 180 mm s^−1 ^ under the voltage of 200 V_p‐p_, which is about 5 times the length of the prototype, and a high speed is realized. However, a dead zone phenomenon exists in the speed of the prototype when the voltage is less than 80 V_p‐p_, this is because the vibration amplitude generated under a voltage of 80 V_p‐p_ cannot overcome the roughness of the marble surface. Meanwhile, the power consumption of the prototype is measured using a digital meter (WT210, Yokogawa, Japan), and the result is shown in Figure 8c. It can be seen that the power consumption becomes larger as the voltage increases. Then, CoT (cost of transportation) can be calculated as

(1) $$\mathrm{CoT}=\frac{P}{mgv}$$

where *P* is the power consumption, *g* is the gravitational acceleration, and *m* and *v* are the weight and speed of the robot, respectively.

The speed and power consumption of the prototype are shown in Figure 12b,c, respectively, and the obtained CoT results are shown in Figure 12d. It can be seen that the CoT is 171.55 at a voltage of 200 V_p‐p_.

**Figure 13:**
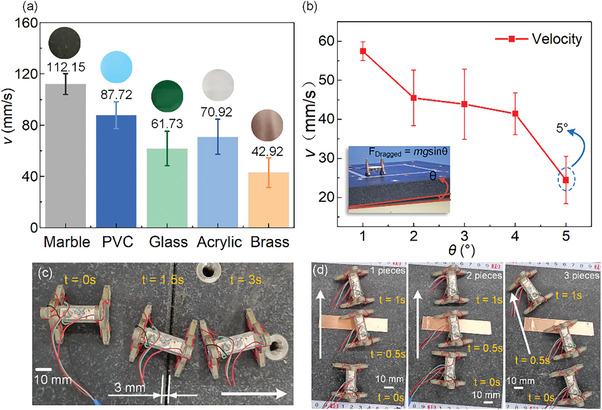
Adaptation experiments of the prototype. a) Motion speed tests on working surfaces with different materials. b) Dragged force tests of the prototype. c) Prototype crossing tests in a 3 mm gully. d) Prototype overcomes the obstacles in 1, 2, and 3 pieces of copper sheets.

To verify the adaptability to different working surfaces, flat working surfaces of different materials are used to test the motion speed first. The voltage amplitude and operating frequency are set as 180 V_p‐p_ and 58.5 kHz. In addition to the previously used marble flat working surface, brass, PVC, acrylic, and glass flat working surfaces are also selected for experimental testing. The test results are shown in **Figure** **13**a, the roughness of the working surface increases from left to right, the prototype has good adaptability on the flat surfaces of different materials, among which the prototype has the fastest motion speed on the flat surface made of marble and the slowest motion on the flat surface made of brass. Large friction can be obtained when the roughness is large, but the increased micro geometric shape error in the vertical direction of the working surface makes it difficult for the foot to cross the distance between peak and valley of the working surface and prevents the robot motion. When the friction is miniature, the robot would “slip” in the process of movement. As shown in Figure 13b, the dragged force is calculated by the climbing marble slope test, in which the voltage and operating frequency are set as 200 V_p‐p_ and 58.5 kHz. The dragged force and the speed are 23 mN and 24.4 mm s^−1 ^ when climbing a slope with an angle of 5°. Besides, the crossing experiment was carried out, as shown in Figure 13c and Movie S2 (Supporting Information). A 3 mm gully was designed in the middle of 2 marble slabs and the prototype could cross it exactly. It is the same as the maximum length of the gully that the prototype could theoretically cross, as the width of the circular unit is 3 mm. Next, the obstacle‐crossing capability was tested by cumulatively stacking copper sheets with a thickness of 0.08 mm on the flat surface until the prototype could not pass. As shown in Figure 13d, it can be seen that the maximum height of the prototype in the experiment is 0.24 mm.

**Figure 14:**
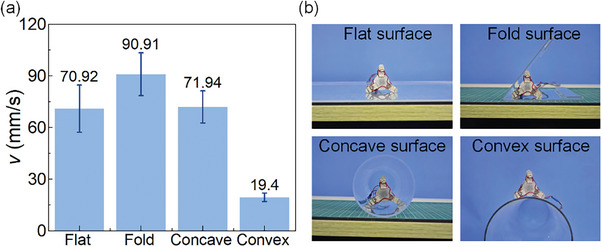
Motion characteristics test on differently shaped working surfaces. a) Speed test results. b) Prototype on different‐shaped working surfaces.

Then the motion characteristics tests on different shaped working surfaces (fold, concave, and convex working surfaces) are performed to further verify the adaptability. As shown in **Figure** **14** and Movie S3 (Supporting Information), the prototype successfully moves on differently shaped working surfaces. Acrylic is selected as the material for different‐shaped working surfaces. The voltage amplitude and operating frequency are set as 180 V_p‐p_ and 58.5 kHz. It can be found that the motion is faster on the fold working surface than on the other working surfaces. This is because the motion speed is related to the contact friction between the foot and the working surface. All 6 feet touch the working surface when the robot moves on the fold working surface, while 4 feet touch the working surface when the robot moves on other working surfaces. When the prototype moves on the convex working surface, the speed is significantly reduced because the angle between the vibration direction of the foot and the working surface and the driving force in the direction of movement are both the minimum.

**Figure 15:**
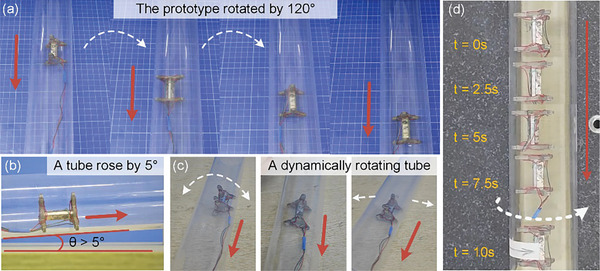
Prototype adaptability tests. a) Movement in the acrylic tube rotated by 120°. b) Movement in the acrylic tube rose by 5°. c) Various disturbances to the prototype during the movement. d) Movement through a rotating acrylic tube. (The red arrow on the figure indicates the motion direction of the prototype, and the white arrow indicates the rotation or movement directions of the acrylic tube).

Finally, the robot motions in a dynamically moving curved surface are investigated (see Movie S4, Supporting Information). The prototype could still move normally in the acrylic tube when the prototype is rotated by 120°, as shown in **Figure** **15**a. The prototype could move in the acrylic tube when the slops dynamically rose by 5°, as shown in Figure 15b. Then, the prototype could still move smoothly when the working surface is disturbed in different ways during the movement, as shown in Figure 15c. The experimental results demonstrate the high adaptability of the designed robot to different working surfaces and its resistance to external disturbances. Moreover, the prototype can move in the rotating acrylic tube, as shown in Figure 15d.

**Table** **3** presents the comparison between this work and previous studies, which shows that the robot can move on different working surfaces while keeping a high speed. It means that the proposed robot has higher adaptability and can adapt to complex and changing terrain environments.

**Table 3:** Comparison between this work and previous studies.

Authors	Length [mm]	Max speed [mm s^−1^]	Working surface	Move in a rotating tube
This work	35.5	179.35	Flat, fold, concave, and convex	Yes
Moon et al.^[^ ^25^ ^]^	/	10.2	Track	No
Fuchiwaki et al.^[^ ^26^ ^]^	32	1.4	Flat	No
Zhong et al.^[^ ^27^ ^]^	10	13.1	Flat	No
Wood et al.^[^ ^30^, ^31^, ^32^, ^33^ ^]^	45	172	Flat	No
Liu et al.^[^ ^34^ ^]^	58	516	Flat, concave	No
Rios et al.,^[^ ^35^, ^36^ ^]^	55	98	Flat	No
Wang et al.^[^ ^37^ ^]^	20	39.3	Flat	No
Li et al.^[^ ^38^ ^]^	67	231.61	Flat	No
Jia et al.^[^ ^39^ ^]^	148.6	136.8	Flat, sand pile	No

## 3 Conclusion

Inspired by the earthworms, a 6‐feet high‐adaptability miniature robot with piezoelectric resonant actuation is developed in this work. The robot adopts the principle of longitudinal‐vibration‐compound actuation to achieve movements on different working surfaces. The robot is designed as a symmetric structure with 3 feet evenly distributed at one Circular unit for high adaptability. Moreover, it is designed as an integrated structure without connectors to achieve the miniature size and lightweight. The structure is optimized by using the finite element method, and the parameters are determined by the modal and transient analyses.

The actuator prototype is fabricated and tested, and its size and weight are only 35.5 × 36.5 × 47, and 22.7 g, miniature size and light weight are achieved. The experiments show that the optimal working frequency is 58.5 kHz and a speed of 179.35 mm s^−1^ is achieved under the voltage of 200 V_p‐p_. The robot can move on flat working surfaces with different materials. In addition, it can move on flat, folded, concave, and convex surfaces, and in a rotating tube through the collaboration of its 6 feet. The experimental results have demonstrated the high adaptability of the proposed miniature robot with piezoelectric resonant actuation to different working surfaces.

In short, this work presented a 6‐feet resonant miniature piezoelectric robot with a symmetrical structure, and the principle of longitudinal‐vibration‐compound actuation with multi‐legged collaboration is designed to improve the working surface adaptability. The experimental results show that the characteristics of high adaptability, miniature size, and light weight are achieved by the developed piezoelectric mobile robot. Our future work will focus on the integrated design with vision sensors and specific exploration applications.

## 4 Experimental Section

### Materials and Fabrication

The prototype consists of a metal base (2A12 duralumin) and 16 PZT sheets (PZT‐4). Figure 9 shows the assembly process of the prototype. The metal base was CNC machined. The surface of the PZT elements was coated with a thin layer and a copper piece was attached to facilitate the excitation signal applied by the solder wire. Then, the prototype was made by gluing the PZT piece to the corresponding position of the metal substrate with epoxy glue. There are 3 steps:

1) Bonding: Apply epoxy resin adhesive evenly on the contact surface of the PZT sheet and leg contact surface.
2) Aging treatment: Arrange the PZT pieces on the leg segments according to the design and clamp them with precision flat pliers; the PZT pieces were completely bonded to the leg segments by epoxy resin after 24 h.
3) Soldering: 1 mm diameter wires were soldered on the outside of the PZT element and used to connect the micro‐robot to the external power supply.

### Measurement of the Vibration Frequency

A scanning laser vibrometer (PSV‐400‐M2, Polytec, Germany) was used to measure the vibration frequency of each unit of the prototype, the scanned area vertically was placed under the scanning head of the instrument, and the vibration speed of the scanned area at different frequencies can be obtained, so as to get the resonance frequency of each unit of the prototype.

### Measurement of the Displacement of the Driving Foot

Two laser displacement sensors (LK‐H020, Keyence) were used to simultaneously collect the signals of the top point of the driving feet along OR_I_ and OZ directions, in which the sampling frequency was set to 392 kHz, and the collected signals were filtered to obtain the displacement curves of the driving feet along OR_I_ and OZ directions, and then synthesize them to obtain the trajectories of the driving feet.

### Measurement of the Speed Performance

Since the prototype moves at a fast speed and over a wide range of motion, the frame superposition method was used to obtain the motion trajectory from the motion movie and calculate the speed. A camera with a frame rate of 30 frames per second was used to record the motion of the prototype. For the speed test, the minimum voltage that can be applied to the motion was first applied, and then the voltage was gradually increased to obtain the motion video of the prototype. In addition, the power consumption was measured by a digital power meter (WT210, Yokogawa, Japan). One point on the robot was selected as a marker to obtain the motion trajectory when processing the motion video, and several motion durations were selected separately to calculate the average velocity.

### Statistical Analysis

Data were expressed as mean ± SD (Standard Deviation). The sample size (n) for each statistical analysis was *n* = 3. Statistical analysis of the data was performed using OriginPro 2021.

## Conflict of Interest

The authors declare no conflict of interest.

## Supporting information

Supplemental Movie 1

Supplemental Movie 2

Supplemental Movie 3

Supplemental Movie 4

## Data Availability

The data that support the findings of this study are available from the corresponding author upon reasonable request.
